# Modification of Biochar Catalyst Using Copper for Enhanced Catalytic Oxidation of VOCs

**DOI:** 10.3390/toxics13060503

**Published:** 2025-06-14

**Authors:** Nan Liu, Jin Zhang, Ya-Lan Cai, Ji-Guo Zhang, Du-Juan Ouyang, Shao-Bo Wang, Qi-Man Xu, Jia-Jun Hu, Di-Ming Chen, Guo-Wen Wang, Ji-Xiang Li

**Affiliations:** 1 Department of Material and Chemical Engineering, Zhengzhou University of Light Industry, Zhengzhou 450001, China; 2 Zhejiang Qiushi Environmental Monitoring Co., Ltd., Hangzhou 310000, China; 3 Shanghai Key Laboratory of Bio-Energy Crops, School of Life Sciences, Shanghai University, Shanghai 200444, China; 4 Yuyao Branch of Ningbo Municipal Bureau of Ecological and Environment, Ningbo 315400, China; 5 Shanghai Advanced Research Institute, Chinese Academy of Sciences, Shanghai 200120, China; 6 University of Chinese Academy of Sciences, Beijing 100049, China

**Keywords:** VOCs, catalytic oxidation technology, copper, biochar

## Abstract

Recently, research has increasingly focused on the introduction of non-precious metals and developing highly stable carriers to enhance catalyst performance. In this study, we successfully synthesized copper (Cu)-modified biochar catalysts utilizing a sequential approach involving enzymatic treatment, liquid impregnation, and activation processes, which effectively enhanced the dispersion and introduction efficiency of Cu onto the biochar, thereby reducing the requisite Cu loading while maintaining high catalytic activity. The experimental results showed that the toluene degradation of 10%Cu@BCL was three times higher than that of unmodified activated carbon (AC) at 290 °C. A more uniform distribution of Cu was obtained by the enzymatic and activation treatments, optimizing the catalyst’s structural properties and reducing the amount of Cu on the biochar. Moreover, the transformation between various oxidation states of Cu (from Cu^0^/Cu(I) to Cu(II)) facilitated the electron transfer during the degradation of toluene. To further understand the catalytic mechanisms, density functional theory (DFT) calculations were employed to elucidate the interactions between toluene molecules and the Cu-modified biochar surface. These findings reveal that the strategic modification of biochar as a carrier not only enhances the dispersion and stability of active metal species but contributes to improved catalytic performance, thereby enhancing its degradation efficiency for VOCs in high-temperature conditions.

## 1. Introduction

At present, volatile organic compounds (VOCs) have emerged as the primary pollutant leading to regional atmospheric pollution. VOC emissions in China are estimated to reach approximately 5 million tons by 2030 [1], with industrial sources contributing about 30% of the total emissions. However, toluene, a widely utilized chemical solvent, is an important component in industrial exhaust gases [2], which are detrimental to air quality and public health. Catalytic oxidation has been recognized as an effective technique for VOC removal due to its high degradation efficiency without generating secondary pollutants [3,4]. The efficacy of catalytic oxidation is heavily reliant on the properties of the catalyst employed. Song et al. found that catalysts could change the electron distribution function and generate active species [5]. Suitable catalysts can improve the degradation efficiency of VOCs [6].

Catalysts typically consists of active substances, carriers, and co-catalysts. Traditionally, precious metals such as platinum are employed as active components due to their low-temperature operational windows and resistance to metal sintering [7]. However, the scarcity and high cost of these metals pose significant economic challenges for industrial applications [8]. In response, studies have focused on developing catalysts utilizing non-precious metal oxides, notably copper (Cu) doping catalysts, which offer cost-effectiveness and commendable catalytic activity in toluene oxidation reactions [9]. Ma et al. demonstrated that a Cu-doped MnO_2_ catalyst exhibits promising application potential due to its high specific surface area and redox properties [10]. Similarly, Xiao et al. found that copper-doped VWTi catalysts show a good toluene degradation rate and selectivity of 99% at 350 °C [11]. Despite these advantages, Cu-based catalysts are prone to sintering or loss during reactions, accelerating the deactivation of active sites and restricting its long-term thermal stability at elevated reaction temperatures [12]. Therefore, it is necessary to find suitable carriers that can uniformly disperse Cu during the loading process and facilitate heat distribution during reactions.

The carrier plays a key role in determining the dispersion of active substances and facilitating efficient mass and energy transfer during reactions [13]. Among various carriers, biochar has extensively been paid attention to for VOC removal due to its advantageous properties, including a large specific surface area [14,15], abundant active sites [16], cost-effectiveness, and sustainable sourcing from diverse biomass feedstocks. Lee et al. [17] highlighted that biochar exhibits a lower energy demand compared to activated carbon. Notably, the activation energy of Ru-Re/biochar for the production of 1,4-Butanediol is three times higher than that of Ru-Re/activated carbon. This can be attributed to biochar’s interconnected network of micropores and mesopores, providing abundant and well-dispersed active sites conducive to the degradation of toluene and other VOCs. Furthermore, other studies have demonstrated that the oxidation state of metals plays a crucial role in activation and degradation of pollutants [18]. Biochar has the capability to reduce the metal oxidation state in homogeneous systems, thereby enhancing the catalytic activity of supported metal catalysts [19]. These findings highlight the versatility and efficacy of biochar as a carrier in the development of catalysts for VOC removal [20,21].

However, the physicochemical properties of biochar are significantly influenced by factors such as feedstock type, preparation methods, and functionalization techniques, which in turn affect its efficacy as a catalyst carrier for toluene degradation [22]. To address these challenges and enhance the performance of biochar-based catalysts, various modification strategies have been explored [23,24,25,26].

Activation temperature: Yorgun et al. observed that increasing the activation temperature from 400 °C to 700 °C led to a sharp decrease in the specific surface area and pore volume of biochar, while augmenting the impregnation ratio of ZnCl_2_ to biochar (1:2–4:1) resulted in increased its specific surface area and pore volume [27]. Similarly, Angin et al. reported that higher activation temperatures (600–900 °C) and impregnation ratios (1:1–4:1) enhanced these textural properties [28]. Notably, biochar activated with a 3:1 ZnCl_2_ ratio for 1 h achieved a surface area of 645 m^2^·g^−1^, a significant increase from the 28 m^2^·g^−1^ of unactivated biochar [29].

Enzymatic treatment: Li et al. discussed the effects of enzyme treatment conditions, including enzyme dosage (180 FPU), treatment duration (60 h), and temperature (57.5 °C) on biochar properties [30]. The findings indicated that enzymatic treatment could alter the structure and crystallinity of raw materials, thereby improving the stability of the resulting biochar.

Chemical impregnation and modifications: The chemical properties and functional groups on the biochar surface are critical for preparing efficient catalysts. It is reported that modifications using agents like Fe_2_O_3_ and MnO_2_ have been shown to enhance the catalytic activity of biochar by improving its pore structure and increasing functional groups, thereby promoting the catalytic conversion of toluene [31].

These strategies suggest the potential of chemical and physical modifications to optimize biochar’s properties as a carrier, thereby enhancing its efficacy in VOC degradation applications [32]. However, the synergistic effects of combining these modification techniques remain underexplored and warrant further investigation to fully understand biochar’s capabilities in catalytic processes.

This study aimed to develop a cost-effective catalyst with high efficiency for the catalytic oxidation of VOCs. Three preparation approaches (enzymatic treatment, liquid impregnation, and activation treatment) were used to modify biochar with Cu. The resulting Cu-modified biochar catalysts were subjected to comprehensive characterization and performance evaluations of reaction activity performance, active site distribution, and surface properties. To elucidate the catalytic oxidation mechanism of Cu-modified biochar in toluene removal, density functional theory calculations were performed. The computational studies provide valuable insights into the interaction between toluene molecules and the catalyst surface, as well as the role of Cu in facilitating oxidation reactions. The findings offer valuable methodologies for optimizing novel catalytic materials on treating organic exhaust gases, emphasizing the potential of preparation methods in enhancing catalytic efficiency under challenging environmental conditions.

## 2. Materials and Methods

### 2.1. Materials

Toluene (C_7_H_8_), an analytical reagent (Ar), was provided by Zhengzhou Pani Chemical Reagent Co. Ltd., (Zhengzhou, China). Copper nitrate (Cu(NO_3_)_2_·3H_2_O, 99.5% Ar) was obtained from Tianjin Kemio Chemical Reagent Co. Ltd. (Tianjin, China). The nitrogen and oxygen was procured from Air Liquide China Holding Co., Ltd. (Shanghai, China). Cellulase was procured from Shanghai Titan Scientific Co., Ltd. (Shanghai, China).

### 2.2. Preparation of Modified Biochar Catalyst Using Copper

In this study, the preparation steps of the catalyst were as follows:

The preparation procedure for Cu-modified biochar catalyst synthesis involved the following steps. (1) Wheat straw (3 g) was crushed and sieved to obtain particles passing through a 40-mesh screen. (2) The sieved biomass was treated with cellulase (10 FPU) and incubated in a shaking flask at 40 °C and 200 rpm for 48 h. (3) The enzymatically treated biomass was immersed in copper nitrate solutions with concentrations ranging from 5% to 25% and shaken at room temperature for 12 h. (4) The impregnated samples were subjected to activation at temperatures between 600 °C and 1000 °C and in a N_2_ atmosphere for 2 h in a tube furnace (OTF-1200X, Kejing, Hefei, China, 50 O.D × 44 I.D × 1000 L mm). The amount of sample obtained was 30% on average.

The catalysts prepared by steps 1, 3, and 4 were recorded as Cu@BC, while those undergoing all four steps were marked as Cu@BCL. In addition, the Cu@BCL samples prepared and activated at 700 °C by 5 °C/min with copper nitrate solutions of 5%, 10%, 15%, 20%, and 25% concentration were recorded as 5%Cu@BCL, 10%Cu@BCL, 15%Cu@BCL, 20%Cu@BCL, and 25%Cu@BCL, respectively. The 10%Cu@BCL samples activated at 600 °C, 700 °C, 800 °C, 900 °C, and 1000 °C are referred to as Cu@BCL-600, Cu@BCL-700, Cu@BCL-800, Cu@BCL-900, and Cu@BCL-1000, respectively.

### 2.3. Catalytic Experiment

**Figure 1:**
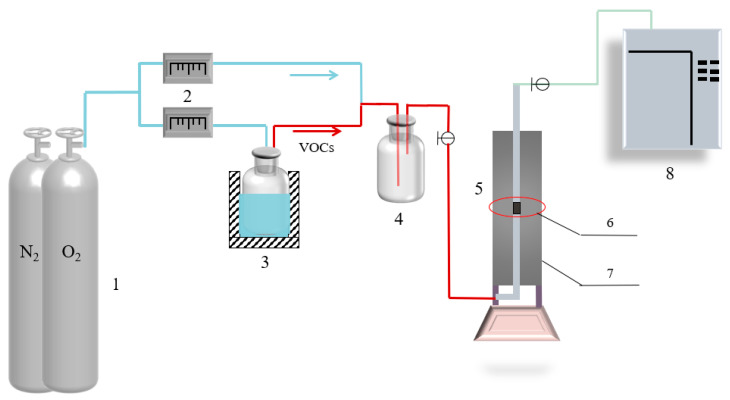
Experimental setup 1. O_2_ and N_2_ gas cylinder. 2. Flow meter. 3. Wash bottle. 4. Gas mixing vessel. 5. Catalytic oxidation reactor. 6. Catalyst filler layer. 7. Insulation jacket. 8. Gas chromatography.

The dynamic catalytic experiment for evaluating the performance of modified biochar catalysts for toluene degradation is illustrated in Figure 1. Nitrogen (N_2_) and oxygen (O_2_) were used to deliver toluene into the catalytic reactor (5). The catalytic reactor (5) housed the biochar catalyst, where the catalytic oxidation of toluene occurred. The toluene inlet concentration was regulated by adjusting the flow rate of N_2_. A gas chromatograph (9) and an intelligent measurement system were used to determinate the toluene concentration and flow rates of gases, respectively. The reaction temperature (T_90_) at which 90% toluene degradation occurred served as an indicator of the evaluation of catalyst performance, and the thermal stability of the catalyst was assessed by measuring the toluene degradation efficiency of the catalyst over a temperature range of 300–500 °C.

Experimental conditions: initial concentration of toluene: 1000 mg·m^−3^; the gas flow rate was evaluated by GHSV (Gas Hourly Space Velocity), and the initial gas flow rate was set to 60,000·h^−1^; concentration of oxygen: 10% (*v*/*v*); gas flow rate of toluene: 0.1 L·min^−1^; temperature range of 230–330 °C; quantity of catalyst: 0.1 g.

### 2.4. Theoretical Calculations

Previous research has systematically summarized the catalyze reaction mechanisms and interfering factors of VOCs [33]. However, the specific effects of Cu on modified biochar during the catalysis process remained inadequately understood. Here, the interaction between Cu-modified biochar and toluene is discussed. We employed first-principles calculations using the Quantum Espresso package (version 7.1) [34]. For optimization of both geometry and lattice size, Brillouin zone integration was performed using 2 × 2 × 1 gamma-centered sampling. To account for the strong correlation effects of metal in the structure, both structural optimizations and electronic structure calculations were carried out using the spin-dependent GGA plus Hubbard correction U method. The adsorption energies of the optimized complexes were calculated by Formula (1) to explore the nature of the intermolecular interaction between biochar modified by Cu and toluene.

(1) $$E_{ads}=E_{complex}-E_{A}-E_{B}$$

where *E_complex_* (kJ·mol^−1^) means the total energy of optimized complex; *E_A_* and *E_B_* (kJ·mol^−1^) denote the energies of biochar modified by Cu and toluene, respectively.

### 2.5. Analysis Method

The concentrations of toluene were determined by a gas chromatograph (GC-7820A, Agilent Technologies, Santa Clara, CA, USA, chromatographic column Agilent 19091N-213, column oven heating program 50 °C for 2 min, then 7 °C/min to 160 °C for 2 min, FID 200 °C). A standard curve of toluene can be seen in SI Figure S1. The microstructures of catalyst samples were indicated by scanning electron microscopy and energy-dispersive X-ray spectroscopy (SEM and EDX, Hitachi Regulus 8100, Tokyo, Japan, Accelerating voltage: 10 kV, magnification: ×10,000 and ×20,000). The surface areas and pore size distributions of the samples were determined using an automatic surface area and aperture analyzer (Mac ASAP2460, Micromeritics Instruments, Norcross, GA, USA, pre-degassing conditions: 120 °C, 6 h, measuring temperature: 77.35 K, gases atmosphere: N_2_). The phase compositions of catalyst samples were measured using an X-ray diffractometer (XRD, Bruker D8 advantage, Bruker Corport, Ettlingen, Germany, 2θ range of 5–90°, scan rate of 2°/min). The elemental composition and chemical state of catalyst samples were characterized by X-ray photoelectron spectroscopy (XPS, ESCALAB 250Xi, Thermo Fisher Technology, Waltham, MA, USA, Scanning range: 0–1350 eV, step size: 1.0 eV), providing insights into oxidation states and surface chemistry. The functional groups of catalyst samples were characterized by Fourier transform infrared spectra (FT-IR, Nicolet IS 5, Thermo Fish Scientific, USA), and 64 scans were performed in 400–4000 cm^−1^ with a resolution of 4 cm^−1^. The thermal stability-related properties of the materials were determined by a thermogravimetric analyzer (TGA, PerkinElmer STA 6000, Springfield, IL, USA, Temperature range: 30–500 °C, heating rate: 10 °C·min^−1^, carrier gas: 10% O_2_/90% N_2_). The amount of metal in the catalyst samples were analyzed by inductively coupled plasma optical emission spectroscopy (ICP-OES, Agilent 5110, Santa Clara, CA, USA, Radiofrequency generator power: 1250 W, pump speed: 60 rpm, Exposure time: 5 s). All data presented in this paper are the mean values of duplicate or triplicate measurements. The confidence level was set at 95%, while the probability of different results was determined by t-distribution.

## 3. Results and Discussion

### 3.1. Study on Preparation Method of Catalyst

#### 3.1.1. Influence of Cu Loading on Catalyst Performance

**Figure 2:**
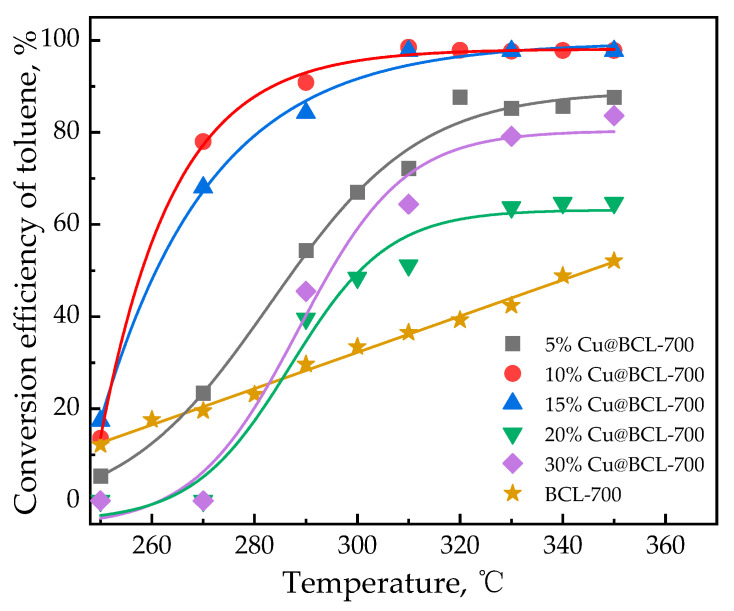
Influence of Cu loading on toluene degradation (activation temperature 700 °C, GHSV 60,000 h^−1^).

To assess the potential for the thermal decomposition of toluene occurring under the experimental conditions, the influence of Cu loading on toluene degradation was investigated. As depicted in Figure 2, the BCL exhibited a toluene conversion rate below 30% at 300 °C. Upon the introduction of Cu into the catalyst, an initial increase in toluene conversion was observed, followed by a decline as the introduction of Cu continued to rise. Notably, catalysts with 10% and 15% Cu loadings achieved approximately 90% toluene degradation at 290 °C, highlighting optimal catalytic performance at these concentrations. However, catalysts with 20% and 30% Cu loadings failed to reach 80% toluene degradation, even during steady-state operation at 340 °C.

#### 3.1.2. Influence of Enzymatic Treatment on Catalyst Performance

**Figure 3:**
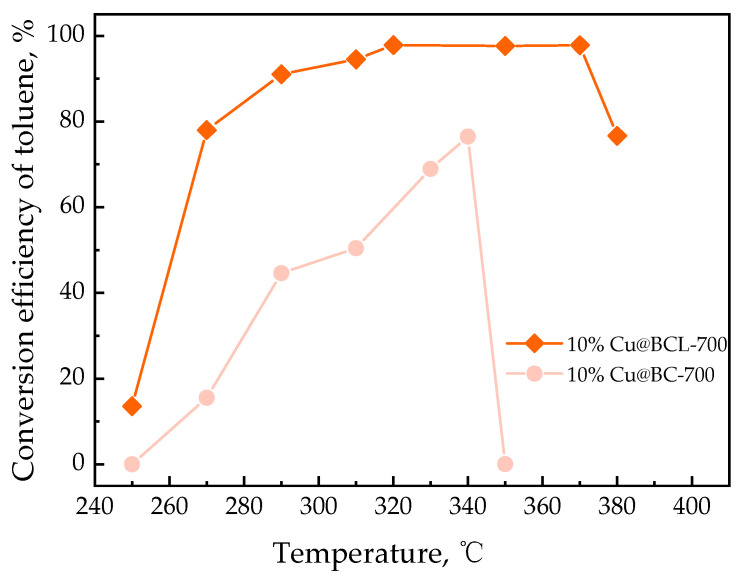
Impact of enzymatic treatment on catalyst performance (10wt% Cu loading, GHSV 60,000 h^−1^).

Figure 3 illustrates the impact of enzymatic treatment on catalyst performance. Comparing the catalytic efficacy of Cu@BC and Cu@BCL, it can be seen that the T_90_ of Cu@BCL decreased from 350 °C for Cu@BC to 290 °C for Cu@BCL, whereas the structure of Cu@BC without enzymatic treatment collapsed at 340 °C before reaching 80% degradation of toluene, suggesting that biochar with enzymatic treatment can maintain structural integrity and superior catalytic activity under similar conditions.

#### 3.1.3. Thermal Stability of Catalytic Materials at Different Reaction Temperatures

**Figure 4:**
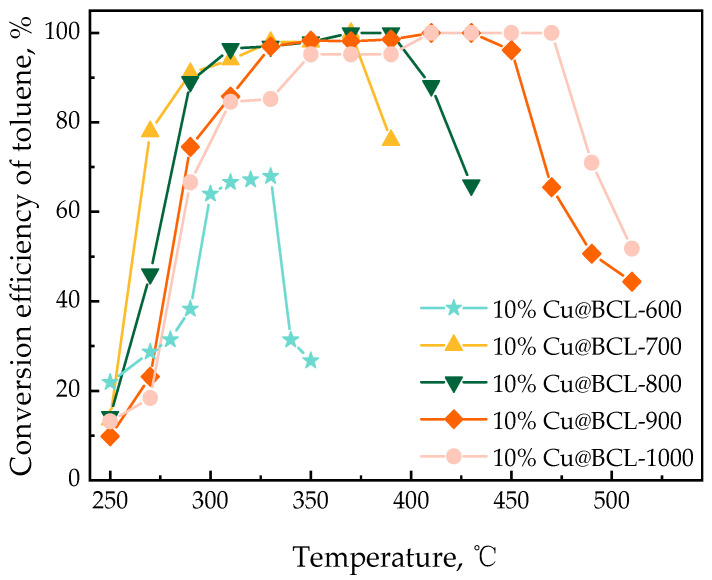
Influence of activation temperature on the degradation efficiency of toluene by different catalysts (GHSV 60,000 h^−1^, temperature range: 30–500 °C, heating rate: 10 °C·min^−1^, carrier gas: 10% O_2_/90% N_2_).

The analysis in the previous section shows that Cu-modified biochar (Cu@BCL) with 10% Cu loading has the best toluene degradation rate. The subsequent studies were carried out on the basis of 10% Cu loading, that is, 10%Cu@BCL is represented by Cu@BCL, on which the catalysts were prepared under varying activation temperatures. As depicted in Figure 4, the degradation efficiency of toluene exhibited a positive correlation with increasing activation temperatures. The T_90_ of both Cu@BCL-700 and Cu@BCL-800 was obtained at 290 °C, indicating superior activity compared to other catalysts. However, the T_90_ of both Cu@BCL-900 and Cu@BCL-1000 required temperatures exceeding 330 °C, and only 65% toluene degradation efficiency of Cu@BCL-600 was achieved at 330 °C. Thermal stability is crucial for the recyclability of catalysts. Above 370 °C, Cu@BCL-700 began to deactivate, whereas Cu@BCL-900 maintained activity up to 470 °C.

**Figure 5:**
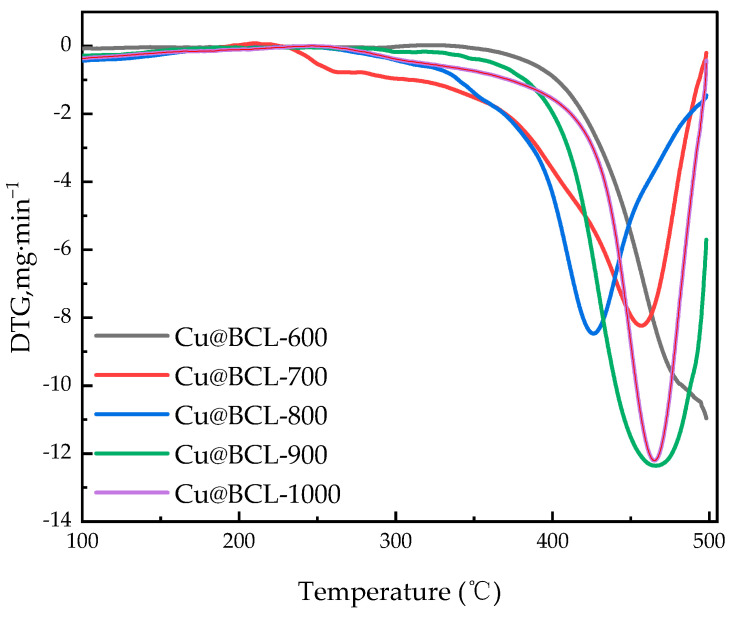
Thermogravimetry of catalytic materials at different reaction temperatures (GHSV 60,000 h^−1^, temperature range: 30–500 °C, heating rate: 10 °C·min^−1^, carrier gas: 10% O_2_/90% N_2_).

This conclusion is also proved by the TG analysis in Figure 5. The initial weight loss of the catalysts was below at 200 °C, attributable primarily to moisture desorption and the removal of loosely adsorbed volatile organic species. A sharper weight was observed between 200 and 400 °C, reflecting the decomposition of organic components and structural changes in the biochar-supported catalyst. At temperatures beyond 500 °C, the rate of mass loss slowed significantly, indicative of the formation of thermally stable carbon frameworks and stable metal oxides. Cu@BCL-700 and Cu@BCL-800 demonstrated higher thermal stability, exhibiting lower mass loss compared to catalysts prepared at higher activation temperatures (e.g., 900–1000 °C).

### 3.2. Effect of Operation Parameters on Catalyst Performance

As mentioned before, GHSV refers to the volumetric flow rate of gas passing through a unit of volume of catalyst per unit of time. It is defined as the ratio of the gas volumetric flow rate to the catalyst bed volume, typically expressed in units of h⁻^1^. GHSV provides a more accurate measure of the contact efficiency between the gas phase and the catalyst, as it directly influences catalyst loading, gas residence time, and economic benefits, thereby serving as a key factor in evaluating industrial reactor performance. As depicted in Table 1, T_90_ exhibited a dependence on the inlet toluene concentration. T_90_ reached its minimum at a toluene concentration of 1500 mg·m^−3^. When the concentration was below 1500 mg·m^−3^, T_90_ increased as the toluene concentration decreased, likely due to a reduced reaction rate under low-concentration conditions. At concentrations exceeding 1500 mg·m^−3^, T_90_ also increased, possibly due to the higher removal load during catalytic oxidation. Nonetheless, the catalyst maintained good activity, with T_90_ variations confined within a 20 °C range, indicating the toluene concentration had little impact on the catalytic activity. It was attributed to the number of active sites available on the surface of the catalyst. On the other hand, due to the shorter residence time of toluene on the catalyst surface under high-GHSV conditions, toluene cannot be in contact fully with the active site.

Table 2 illustrates the impact of GHSV on toluene degradation efficiency at a reaction temperature of 300 °C. The optimal degradation efficiency was observed at a GHSV of 60,000 h^−1^, which was 11% higher than that at a GHSV of 90,000 h^−1^. This suggests that higher GHSVs, corresponding to shorter residence times, may limit the contact between toluene molecules and active sites on the catalyst surface, thereby reducing degradation efficiency. At low space velocities, the residence time of reactants is prolonged. At a fixed temperature, the number of active sites activated on the surface of the catalyst is constant. Excessive surface coverage leads to the blockage of active sites. Overall, Cu@BCL demonstrated excellent adaptability at high GHSVs under low-temperature catalytic conditions. Although a decrease in toluene degradation efficiency was observed at elevated GHSVs, the catalyst still shows promise for future industrial applications.

### 3.3. Characterization

#### 3.3.1. Surface Structure Analysis

**Figure 6:**
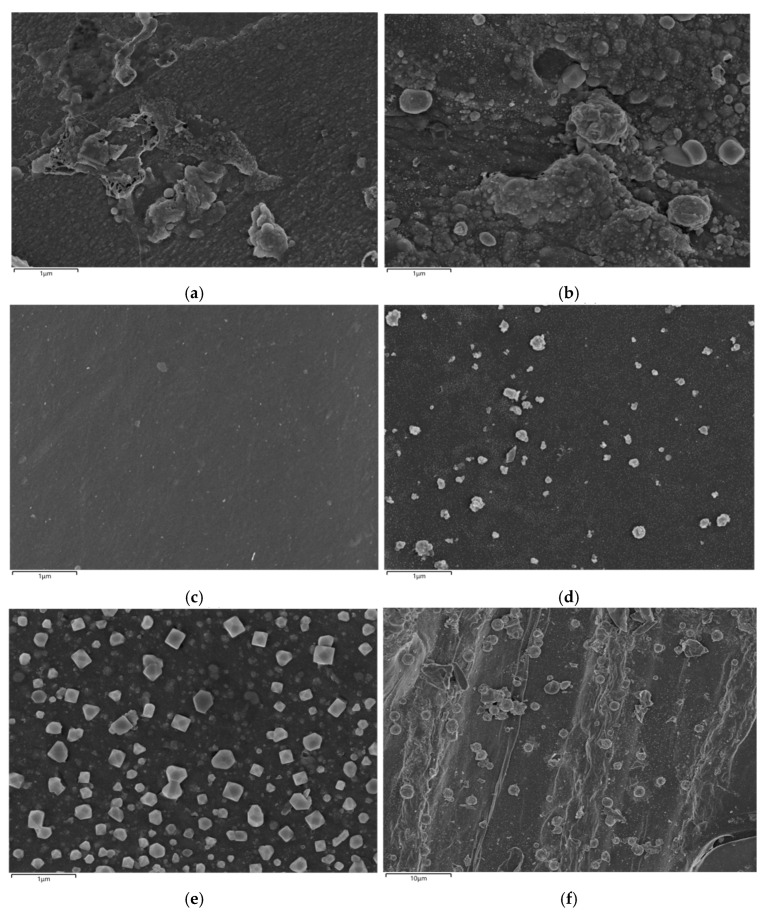
SEM images of catalysts prepared under different conditions: (**a**) BC-700; (**b**) Cu@BC-700; (**c**) BCL-700; (**d**) Cu@BCL-700; (**e**) Cu@BCL-800; (**f**) Cu@BCL-1000 (accelerating voltage: 10 kV, magnification: ×10,000 and ×20,000).

Figure 6a–f present SEM images of catalysts prepared under different conditions. It can be seen from Figure 6a that the untreated biochar exhibits a rough surface with visible impurities. These impurities are markedly reduced after enzymatic treatment, resulting in a cleaner surface. In Figure 6b, the Cu@BC catalyst displays noticeable agglomeration on its surface. Conversely, Figure 6c–f, depicting enzymatically treated catalysts, reveal smoother surfaces with well-dispersed copper particles. In addition, SI Figure S3 illustrates that the N_2_ adsorption–desorption isotherms of the catalysts presented in Table 3 showed characteristics of Type I isotherms, indicative of materials mainly possessing microporous structures. Furthermore, it can be seen from Table 3 that a decrease in the specific surface area of the biochar is observed after enzymatic treatment, accompanied by an increase in pore size. Notably, the pore size of Cu@BCL-1000 is 0.19 nm larger than that of Cu@BCL-700, and its specific surface area is enhanced by 102 m^2^·g^−1^. Furthermore, the T_90_ value for Cu@BCL-1000 is 20 °C higher than that of Cu@BCL-700.

#### 3.3.2. XRD Analysis

**Figure 7:**
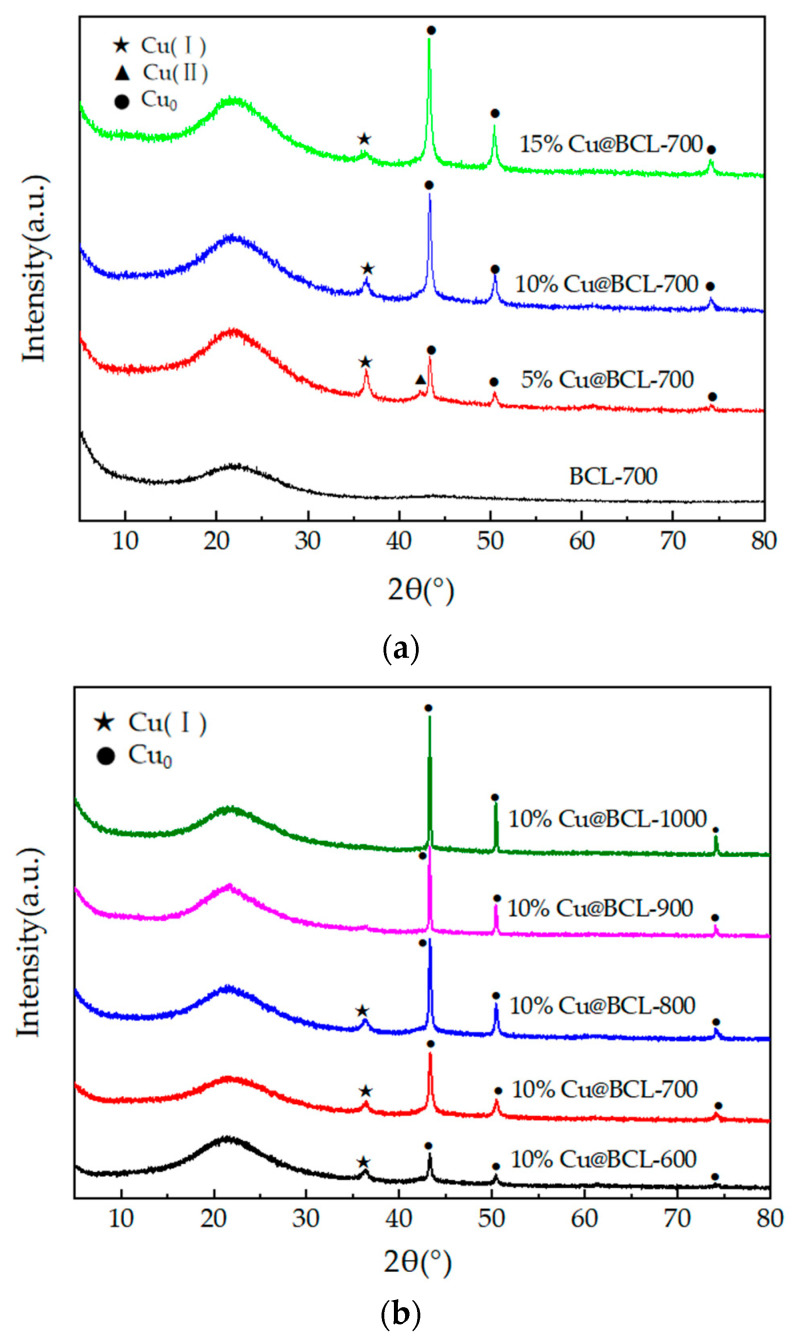
XRD patterns of catalysts prepared under different conditions. (**a**) XRD patterns of catalysts with varying Cu loadings. (**b**) XRD patterns of catalysts subjected to different activation temperatures (2θ range of 5–90°, scan rate of 2°/min).

Figure 7a shows the XRD patterns of catalysts with varying Cu loadings. The original biochar exhibited an amorphous structure, as evidenced by the absence of distinct diffraction peaks. Upon Cu loading, prominent diffraction peaks emerge at 2θ angles of 43.3°, 50.4°, and 74.1° (black dot shown in Figure 7a), corresponding to the (111), (200), and (220) crystallographic planes of Cu^0^ (PDF-# 04-0836), respectively. Among these, the (111) plane at 43.3° displays the highest intensity. As the Cu loading increases, the intensities of these Cu^0^ peaks increase accordingly. Meanwhile, minor diffraction peaks appear at 36.4° and 42.3° (2θ, black star and triangle shown in Figure 7a, respectively), aligned with the (111) crystal planes of Cu(I) (Cu_2_O) and Cu(II) (CuO) (PDF-# 05-0667). However, the relative intensities of these oxide peaks diminish with increasing Cu loading. Figure 7b illustrates the XRD patterns of catalysts subjected to different activation temperatures. Under the same loading amount of Cu, elevating the activation temperature leads to intensified diffraction peaks of Cu^0^. Conversely, the diffraction peaks of Cu(I) (Cu_2_O) and Cu(II) (CuO) became even weaker along with increasing activation temperatures [35].

#### 3.3.3. XPS Analysis

**Figure 8:**
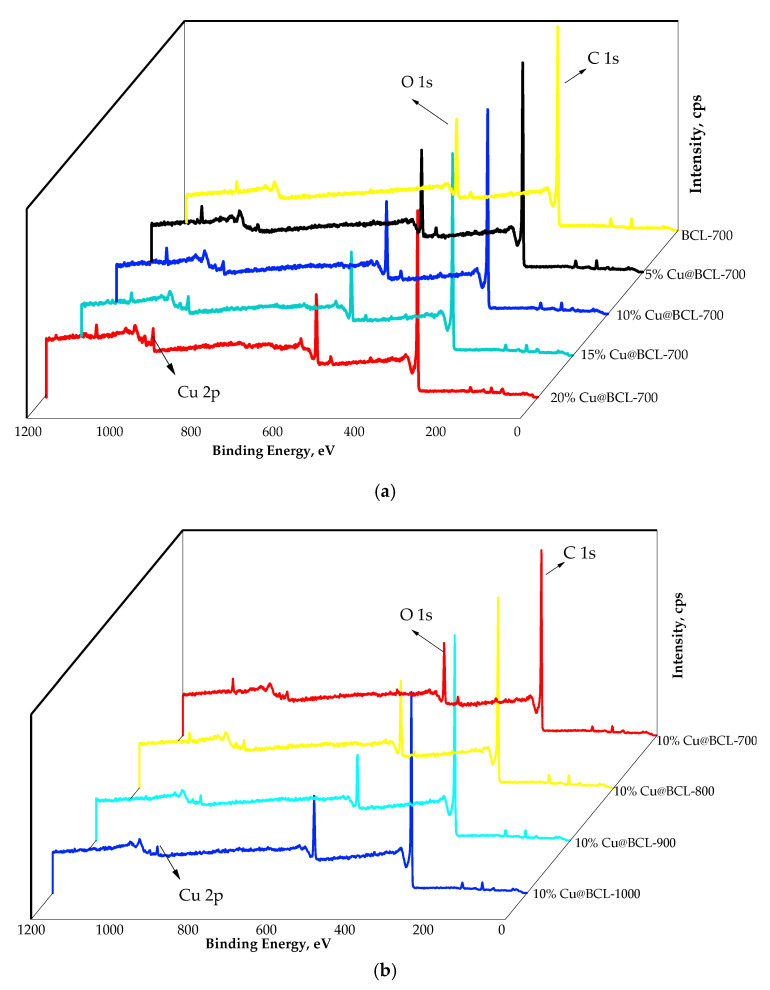
XPS patterns of catalysts prepared under different conditions. (**a**) XPS patterns of catalysts with varying Cu loadings. (**b**) XPS patterns of catalysts with varying activation temperatures. (Scanning range: 0–1350 eV, step size: 1.0 eV.)

The chemical valence analysis of Cu@BCL catalysts is shown in Figure 8. It reveals distinct peaks corresponding to Cu, oxygen (O), and carbon (C) elements, with no detectable impurities. The binding energies align with those characteristic of Cu, O, and C, confirming the elemental composition of the catalyst.

**Figure 9:**
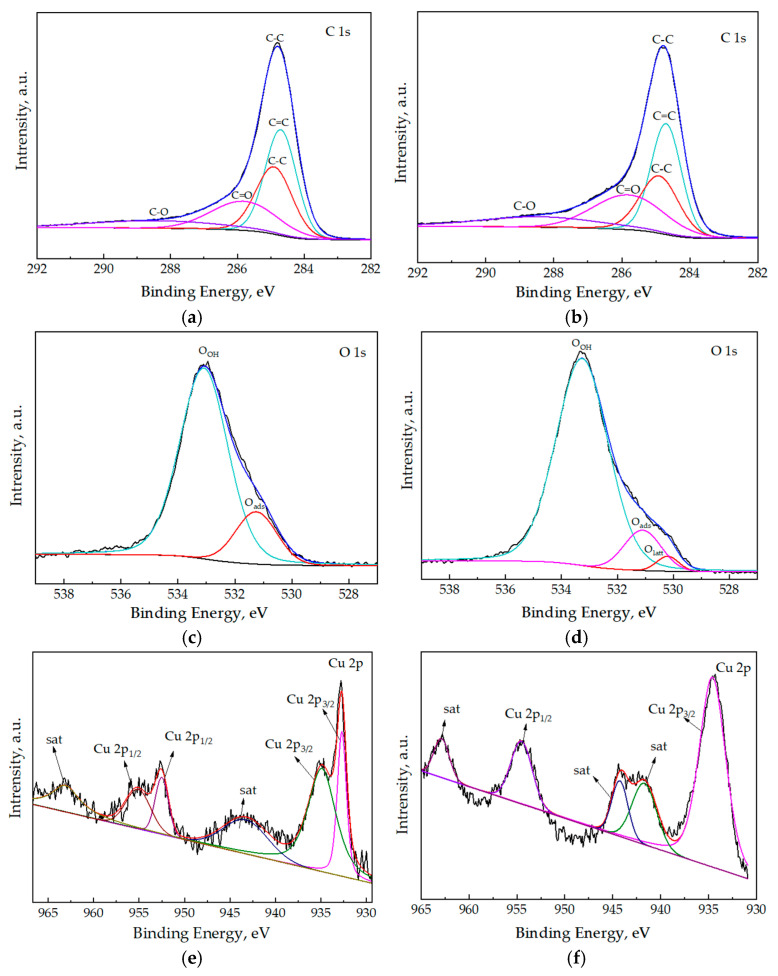
The chemical valence analysis of Cu@BCL catalyst. (**a**) C 1s before catalyst reaction; (**b**) C 1s after catalyst reaction; (**c**) O 1s before catalyst reaction; (**d**) O 1s after catalyst reaction; (**e**) Cu 2p before catalyst reaction; (**f**) Cu 2p after catalyst reaction. (Scanning range: 0–1350 eV, step size: 1.0 eV.)

Figure 9a,b are C 1s spectra before and after the Cu@BCL reaction. The observed peaks at binding energies of 284.7 eV, 284.9 eV, 285.8 eV, and 288.4 eV are attributed to the C=C, C–C, C=O, and C–O functional groups, respectively [36]. The O 1s spectra, shown in Figure 9c,d, exhibit three characteristic peaks at 533.3eV (O_3_), 531.1eV (O_2_), and 530.2 eV (O_1_). These peaks are typically associated with different oxygen species, such as hydroxyl groups, adsorbed oxygen, and lattice oxygen, respectively [37]. Figure 9e,f are Cu 2p spectra before and after Cu@BCL reaction. The main peaks at 952.5 eV (Cu 2p_1/2_) and 934.6 eV (Cu 2p_3/2_) indicate the presence of Cu^0^ species in the catalyst prior to the reaction [38]. Additionally, secondary peaks at 954.5 eV and 932.7 eV, with a binding energy difference of 19.7 eV, along with satellite peaks at 941.7 eV and 963.1 eV, clearly suggest the existence of Cu(I) and Cu(II). These satellite features are characteristic of Cu oxide species and are commonly used to distinguish between different copper oxidation states [39].

#### 3.3.4. Functional Group Analysis

**Figure 10:**
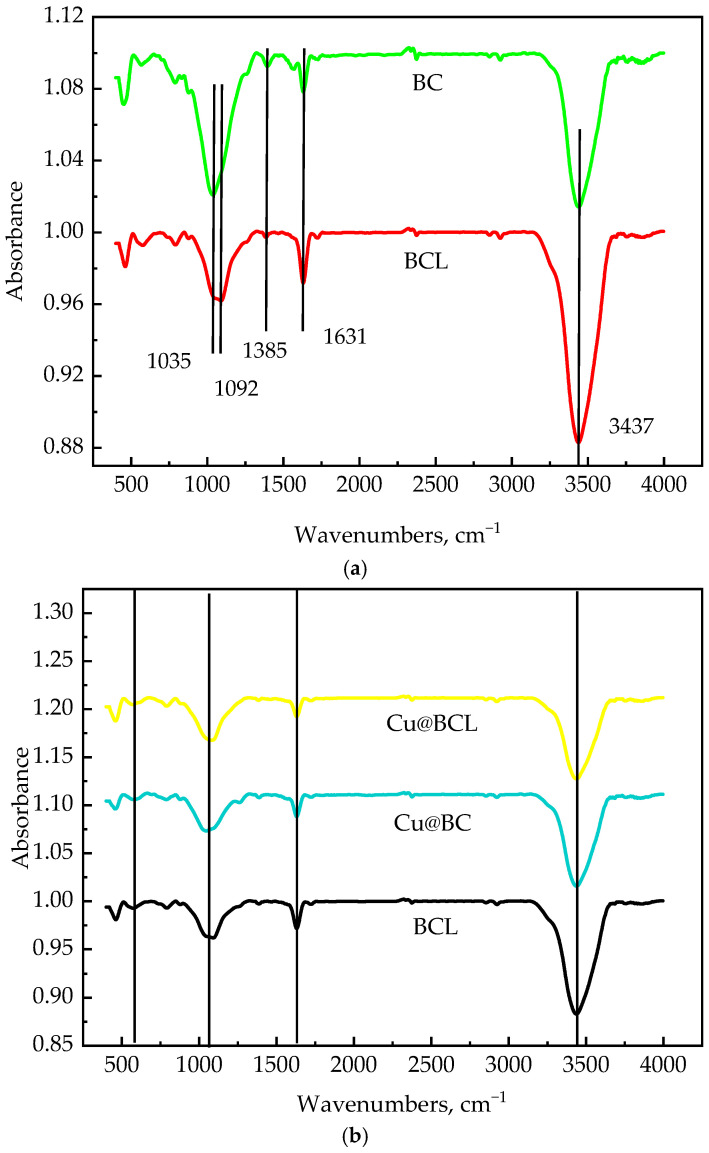
The FT-IR spectra of catalysts prepared under different conditions. (**a**) The FT-IR spectra of BC and BCL; ■ BC; ■ BCL. (**b**) FT-IR spectra of BCL, Cu@BC, and Cu@BCL; ■ BCL; ■ Cu@BC; ■ Cu@BCL (64 scans in 400–4000 cm^−1^, resolution of 4 cm^−1^).

The FTIR spectra of Cu-modified biochar catalysts, as illustrated in Figure 10, reveal distinct functional groups associated with their chemical structure. An absorption band at 1630.56 cm^−1^ corresponds to the stretching vibrations of C=C bonds, while the stretching vibration peaks of the C-O bonds are evident at 1091.52 cm^−1^ [40]. As shown in Figure 10b, catalysts with enzymatic treatment contain fewer C-O functional groups, and Cu@BCL, demonstrating superior catalytic performance, shows the lowest intensity in the C–O absorption band. Furthermore, the emergence of a new absorption peak at 570 cm^−1^ in the Cu@BCL spectrum signifies the formation of Cu–O bonds, indicative of copper oxide species on the biochar surface [41].

### 3.4. Macroscopic Catalytic Mechanism

The degradation efficiency of VOCs via catalytic oxidation is influenced by various factors, including temperature fluctuations and humidity variations inherent in industrial exhaust gases [42]. Ensuring the efficacy and stability of catalysts under these conditions is crucial, with the performance being significantly affected by the choice of raw materials and preparation methods [43]. A Cu-modified biochar catalyst was successfully synthesized by leveraging the abundant active sites and cost-effectiveness of the biochar with the catalytic properties and economic advantages of Cu over precious metals. The key factors and underlying mechanisms involved in the preparation of the Cu-modified biochar catalyst were systematically investigated.

The introduction of Cu into biochar catalysts significantly influences their structural and electronic properties, facilitating the oxidation state cycling and electron transfer processes, thereby enhancing their catalytic performance in toluene oxidation. For biochar without copper introduction (BCL, Figure 2), under reaction conditions of 300 °C, the outlet of the toluene concentration was equal to 70% of the inlet toluene concentration, indicating that the observed toluene reduction was primarily due to adsorption rather than catalytic degradation. Combined with the results of the loading amount of Cu on toluene degradation, the degradation of toluene at high temperatures was shown to be mainly caused by the catalyst with active components. XRD analysis has revealed that the original biochar lacked a defined crystal structure. Upon loading with active copper species, distinct diffraction peaks corresponding to Cu^0^, Cu(I), and Cu(II) were observed, indicating the formation of an oxide layer on the biochar surface. As the Cu loading increased, the intensity of the Cu^0^ diffraction peaks correspondingly increased, while the peaks associated with Cu(I) and Cu(II) diminished. This finding suggests that higher Cu loading favors the formation of metallic Cu, which is known for its significant catalytic activity [44]. The presence of Cu(I) exhibited superior carrier mobility, thereby markedly enhancing oxidation state cycling and electron transfer processes within the biochar matrix. After the reaction, the phase composition of the Cu-modified biochar remained stable, maintaining optimal catalytic activity and reliability.

The characteristic peak at 533.3 eV (O_3_) in the O 1s XPS spectrum corresponds to groups such as C–OH and C=O, confirming the successful introduction of these functionalities (Figure 9a). These groups enhance biochar’s ability to interact with toluene molecules, facilitating effective degradation. As indicated in Table 3, increasing Cu loading led to a gradual decrease in both the specific surface area and porosity of the biochar. This trend suggests that Cu particles occupy the pores of the biochar, reducing its overall surface area. Such pore blockage also hinder the access of toluene molecules to active sites. However, the catalytic activity for toluene degradation exhibited a dependence on Cu loading. Biochar samples with 10% and 15% Cu loadings demonstrate superior performance. In contrast, low Cu loadings (<10%) may underutilize the biochar surface. Higher Cu loadings (20% and 30%) failed to reach 80% degradation even at 340 °C, indicating that excessive Cu loadings (>15%) may lead to agglomeration, reducing the dispersion of active substances and catalytic efficiency. These findings underscore the importance of optimizing Cu loading to balance the availability of active sites and the surface structure of the biochar. Moderate Cu loadings ensure sufficient active sites while maintaining the porous structure necessary for effective toluene adsorption and degradation.

Enzymatic treatment had a key role in enhancing the structural and catalytic properties of Cu-modified biochar catalysts in the current study. SEM analyses revealed that the biochar surface was characterized by roughness and the presence of impurities before enzymatic treatment, while these impurities were significantly reduced after enzyme treatment. Although this enzymatic treatment leads to a decrease in specific surface area and total pore volume, as indicated in Table 3, the effective area and reaction space for Cu integration was concurrently enhanced. The smoother surfaces observed in Figure 6e,f show that post-enzymatic treatment allows for clearer visualization and integration of Cu and Cu oxides into the carbon framework. The EDX results in SI Figure 2 also confirm the absence of copper in pure biochar samples (BC-700, BCL-700), while clear copper signals are detectable for Cu-loaded catalysts. Notably, enzymatic treatment (e.g., 10%Cu@BCL-700) significantly enhances the uniformity of copper dispersion, providing smoother surfaces and more homogeneous metal distribution. This structural refinement improved catalytic performance. Notably, the Cu@BCL (Figure 3) catalyst achieved a T_90_ at 290 °C, whereas the non-enzymatically treated Cu@BC’s structure collapsed at 340 °C before reaching 80% toluene degradation. This conclusion is also verified by ICP elemental analysis (SI Table S2). Enzymatic treatment in 10%Cu@BCL-700 reduced Cu content (14.94%) but significantly improved dispersion. The balance between copper loading and uniformity was optimal here, potentially enhancing catalytic performance due to more effective utilization of copper active sites. Activation temperatures above 700 °C increased Cu content (21.72% at 1000 °C) but concurrently reduced dispersion uniformity and catalyst stability, as evidenced by the EDX results in SI Figure S2. The higher Cu content at elevated temperatures resulted from agglomeration and sintering phenomena that reduced the volatility of Cu species. Furthermore, enzymatically treated catalysts exhibited a reduction in C–O functional groups and an increase in C=C and C=O groups, as evidenced in Figure 9b,d. This alteration suggests decreased oxygen content on the biochar surface, generating additional active sites that enhance Cu loading efficiency. This finding confirms that enzymatic treatment not only promotes uniform Cu dispersion but also significantly enhances toluene degradation efficiency.

The activation temperature exerted a multifaceted influence on the structural characteristics and catalytic performance of porous biochar, particularly in the context of oxygen reduction reactions. High temperatures facilitate the development of a graphitized carbon framework, altering the forms of oxygen and Cu present on the biochar surface. These modifications significantly influenced material adsorption properties and catalytic efficacy. As depicted in Figure 4 and the TGA results in Figure 5, the T_90_ of Cu@BCL-700 and Cu@BCL-800 was obtained at 290 °C, indicating superior activity compared to other catalysts. However, Cu@BCL-700 began to deactivate beyond 370 °C. In practical industrial applications, reactors often experience uneven temperature distributions, making it crucial to consider the thermal stability of catalysts under such conditions. Table 3 reveals that Cu@BCL-700 and Cu@BCL-1000 exhibited the highest specific surface areas and pore sizes. This observation suggested that activation at elevated temperatures alleviated pore blockage caused by the introduction of Cu. The specific surface area and pore size of the catalyst are critical factors influencing its thermal resistance and stability of catalysis [45]. Nonetheless, high activation temperatures (800 °C, 1000 °C) lead to the visible agglomeration of copper, which can negatively affect the dispersion quality, as evidenced in SI Figure S2e,f. However, the crystallite sizes in the catalyst are subjected to different activation temperatures, as shown in SI Table S1. With the same Cu loading, increasing the activation temperature does not cause significant changes in the crystallite size of Cu, indicating that the increase in activation temperature does not obviously affect the dispersion state and morphology of Cu, but there is potential over-fracturing of organic chains within the catalyst, that is, metal sintering [46]. Such structural degradation is detrimental to the adsorption and mass transfer of toluene on biochar, thereby impairing its degradation efficiency. Although the T_90_ of Cu@BCL-900 and Cu@BCL-1000 was achieved exceeding 330 °C, the deactivation temperature of Cu@BCL-1000 reached 470 °C. Furthermore, with increasing activation temperatures, there was a notable reduction in oxygen-containing elements (H, N, O) within the biochar. Single-bonded oxygen (C–O) groups decreased, while double-bonded oxygen (C=O) become more prevalent, leading to an increased presence of C=C and C=O groups, as illustrated in Figure 9b and Figure 10. Concurrently, the carbon content rose significantly, resulting in decreased N/C and H/C ratios. It has been widely reported that biochar can reduce surface-bound metal ions during pyrolysis [19]. Consequently, higher activation temperatures favor the formation of Cu^0^ on the biochar surface, while the concentrations of Cu(I) and Cu(II) reduce, as shown in Figure 7b. This structural evolution made by higher activation temperatures enhances the introduction of Cu^0^, thereby improving biochar heat resistance and mechanical strength [47]. The stability of biochar was effectively enhanced.

Analyzing the chemical valence of the Cu@BCL catalyst before and after the reaction confirmed the exclusive presence of Cu, O, and C elements, with no detectable impurities (Figure 8). This finding indicated the successful synthesis of Cu sites on the biochar surface, providing abundant active sites for catalytic processes. The absence of a characteristic peak at 530.2 eV (O_1_) corresponded to O in metal oxides, alongside the presence of a peak at 531.1 eV (O_2_) and associated with coordinatively unsaturated oxygen, suggesting the formation of numerous active defect sites within the Cu@BCL catalyst (Figure 9c,d). Moreover, the proportion of O_2_ (531.1 eV) decreased while O_1_ (530.2 eV) increased, indicating that the partial oxidation of Cu species was converted into Cu ions within the catalyst. The surface activation mechanism revealed that solid-state Cu(II) exhibited significantly higher catalytic activity compared to Cu(II) ions (Cu^2+^) [48]. The binding energy shifts observed in Figure 9e,f confirmed the oxidation of Cu^0^ and Cu(I) to Cu(II) on the Cu@BCL surface throughout the catalytic reaction. This interconversion among various oxidation states of Cu (from Cu^0^/Cu (I) to Cu (II)) facilitated electron transfer during toluene degradation. Additionally, the inevitable oxidation of Cu^0^ and Cu(I) to Cu(II) upon air exposure prior to the reaction aligned with XRD analysis results [49,50].

**Figure 11:**
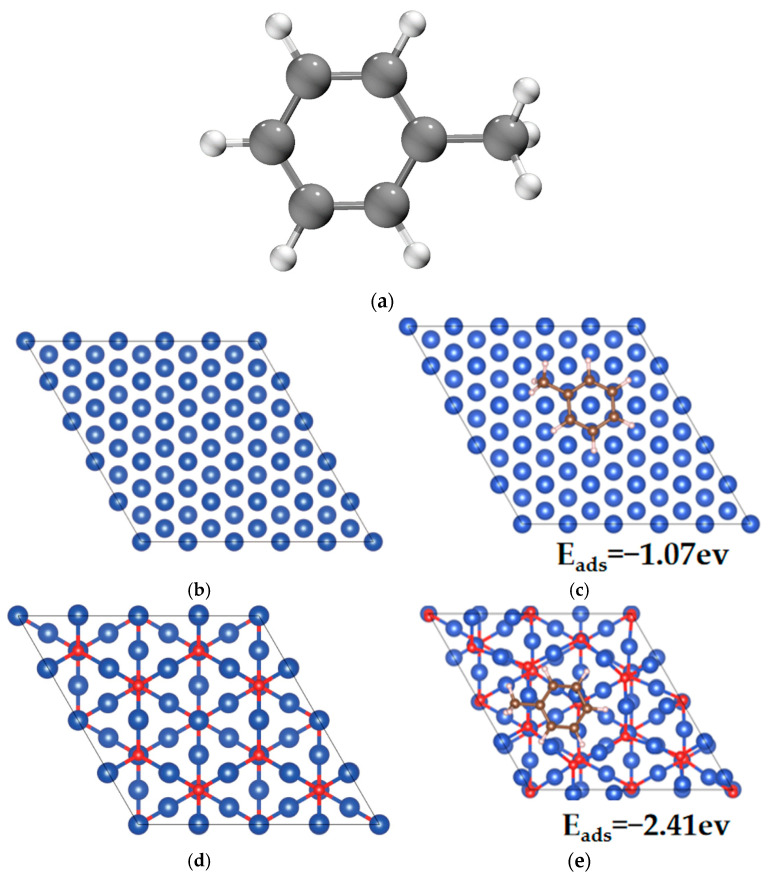
Visualizations of interactions between toluene and Cu@BCL. (**a**) Geometry optimizations of toluene. (**b**) Geometry optimizations of Cu (111). (**c**) Interactions between toluene and Cu (111). (**d**) Geometry optimizations of Cu_2_O (111). (**e**) Interactions between toluene and Cu_2_O (111).

The diffraction patterns presented in Figure 7 indicate that the 111 crystal planes of Cu^0^, Cu(I) [Cu_2_O], and Cu(II) [CuO] exhibited the most pronounced intensities among the observed crystallographic orientations. Adsorption energies were calculated for toluene on both Cu^0^(111) and Cu_2_O(111) surfaces to elucidate the interaction between copper species on biochar and toluene. The adsorption energy on the Cu^0^(111) surface was −1.07 eV, indicating a spontaneous adsorption process, which implied a stable interaction between toluene and the metallic Cu surface (Figure 11). The adsorption energy on the Cu_2_O(111) surface was significantly more negative, at −2.41 eV, suggesting more stable adsorption and a stronger interaction between toluene and the Cu (I) oxide surface. This enhanced stability was closely associated with the distinct chemical characteristics of the Cu_2_O(111) surface. The presence of a coordinatively unsaturated Cu(I) on this surface created stronger active centers through metal–O synergistic sites, thereby enhancing the adsorption and degradation capacity for toluene. Consequently, catalysts such as Cu@BCL-700 and Cu@BCL-800, which likely possess a higher proportion of these active sites, exhibited superior activity compared to catalysts prepared at higher activation temperatures.

To summarize, the catalytic performance variations observed in the biochar-based catalysts were predominantly attributed to differences in their physical structures, influenced by the key factors of Cu loading amount, enzymatic treatment, and activation temperature. Optimizing these parameters is crucial for enhancing toluene degradation efficiency. Therefore, the Cu@BCL-700 catalyst demonstrated superior catalytic performance due to the above-mentioned factors, which collectively enhanced the density and dispersion of active sites, reactivity, and effective utilization of the biochar pore structure, leading to efficient toluene degradation.

## 4. Conclusions

A Cu-modified biochar was successfully synthesized through enzyme and activation treatment in the current study to enhance toluene degradation capacity at relatively low temperatures of catalytic oxidation. The 90% toluene adsorption efficiency of Cu@BCL-700 appeared at the lowest temperature of 290 °C. The introduction of Cu enhanced catalytic efficiency through its influence on the surface structure of the catalysts. Moreover, enzymatic treatment effectively removed surface impurities, facilitating uniform Cu dispersion and increasing the effective surface area. Higher activation temperatures improved biochar structure, altering the forms of O and Cu on the surface, thereby enhancing thermal resistance and stability. The catalytic mechanism involves the interconversion of Cu oxidation states, promoting electron transfer during toluene degradation. Toluene adsorption is more stable on the Cu(I) surface, providing stronger active centers through metal–oxygen synergistic sites, thus significantly enhancing adsorption and degradation capabilities. Consequently, Cu@BCL-700 and Cu@BCL-800 exhibited superior activity compared to catalysts prepared at higher activation temperatures. This study presents an efficient method for preparing biochar-based catalysts, offering an optimized solution for the effective removal of toluene-containing exhaust gases.

## Figures and Tables

**Table 1:** Toluene concentration on the catalytic performance of 10%Cu@BCL-700 catalysts.

Toluene Concentration, mg·m^−3^	500	1000	1500	2000	2500	3000
T_90_, °C	290	285	280	290	295	300

Reaction temperature: 300 °C.

**Table 2:** GHSV on the catalytic performance of 10%Cu@BCL-700 catalysts.

GHSV, h^−1^	90,000	75,000	60,000	45,000	30,000
Degradation rate of toluene, %	84.32	89.09	95.28	88.63	83.97

Reaction temperature: 300 °C.

**Table 3:** Pore structure parameters of catalysts prepared under different conditions.

Sample	Specific Surface Area S_BET_ (m^2^·g^−1^)	Total Pore Volume (cm^2^·g^−1^)	Micropore Volume (cm^2^·g^−1^)	Mean Aperture (nm)
BC	254	0.15	0.11	2.4
BCL	216	0.13	0.09	2.4
5%Cu@BCL	209	0.12	0.10	2.4
10%Cu@BCL	200	0.11	0.09	2.3
20%Cu@BCL	170	0.10	0.08	2.3
30%Cu@BCL	153	0.09	0.07	2.3
Cu@BCL-1000	302	0.19	0.12	2.5

## Data Availability

The data used to support the findings of this study are currently under embargo. Requests for data, 6 months after publication of this article, will be considered to upload to the repository of FIGSHARE.

## Supplementary Materials

The following supporting information can be downloaded at: https://www.mdpi.com/article/10.3390/toxics13060503/s1, SI Figure S1. Standard curve of Toluene by GC (column oven heating program 50 °C for 2 min, then 7 °C/min to 160 °C for 2 min, FID 200 °C). SI Figure S2. SEM images and EDX images of catalysts prepared under different conditions (a) 10%BC-700; (b) 10%Cu@BC-700; (c) 10%BCL-700; (d) 10%Cu@BCL-700; (e) 10%Cu@BCL-800; (f) 10%Cu@BCL-1000 (Accelerating voltage: 10 kV, magnification: ×10,000 and ×20,000). SI Figure S3. The isotherms of N_2_ adsorption-desorption for different samples. (Initial temperature: 77.35 K, Carrier gas: N_2_). SI Table S1. Crystallite size of catalysts subjected to different activation temperatures. SI Table S2. ICP parameters of the catalysts prepared under different conditions. (Radiofrequency generator power: 1250 W, pump speed: 60 rpm, Exposure time: 5 s).
