# Supplementary material for: Modification of Biochar Catalyst Using Copper for Enhanced Catalytic Oxidation of VOCs
Source: Toxics. 2025 Jun 14;13(6):503. doi: 10.3390/toxics13060503 (PMC12197257; doi:10.3390/toxics13060503)
Supplement: Supplementary file 1 [file toxics-13-00503-s001.zip › toxics-3583942-supplementary.pdf]

SI Figure S1 Standard curve of Toluene by GC (column oven heating program 50°C for 2 minutes, then 7°C/min to 160°C for 2 minutes, FID 200°C)

SI Figure S2 SEM images and EDX images of catalysts prepared under different conditions (a) 10%BC-700; (b) 10%Cu@BC-700; (c) 10%BCL-700; (d) 10%Cu@BCL-700; (e) 10%Cu@BCL-800; (f) 10%Cu@BCL-1000 (Accelerating voltage: 10 kV, magnification: ×10,000 and ×20,000)

SI Figure S3 The isotherms of N_2 adsorption-desorption for different samples. (Initial temperature: 77.35 K, Carrier gas: N_2)

SI Table S1 Crystallite size of catalysts subjected to different activation temperatures

SI Table S2 ICP parameters of the catalysts prepared under different conditions. (Radiofrequency generator power: 1250W, pump speed: 60rpm, Exposure time: 5s)

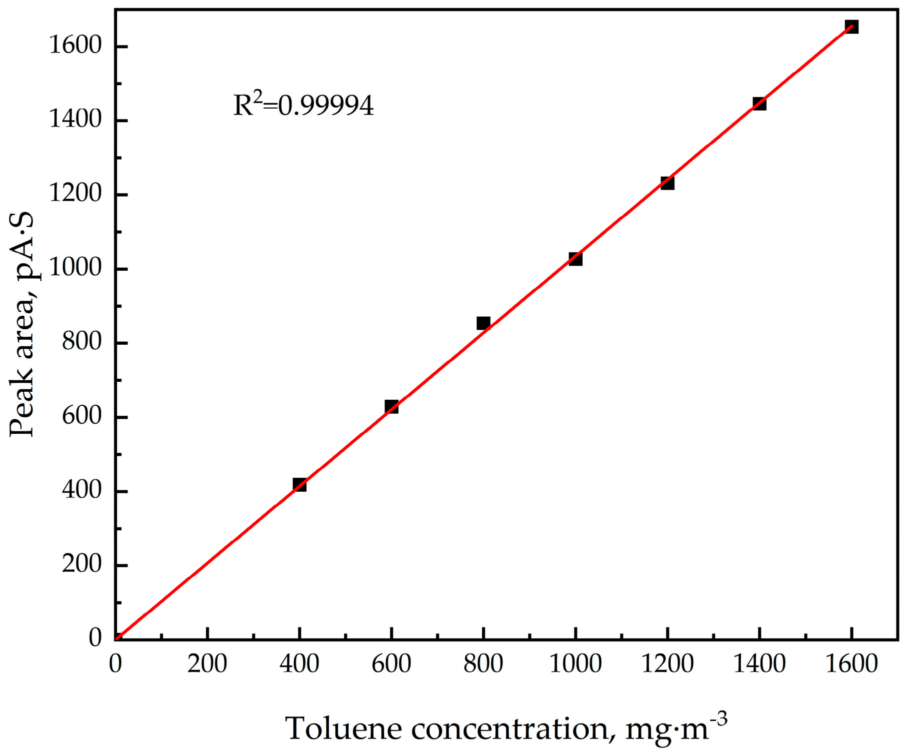

SI Figure S1

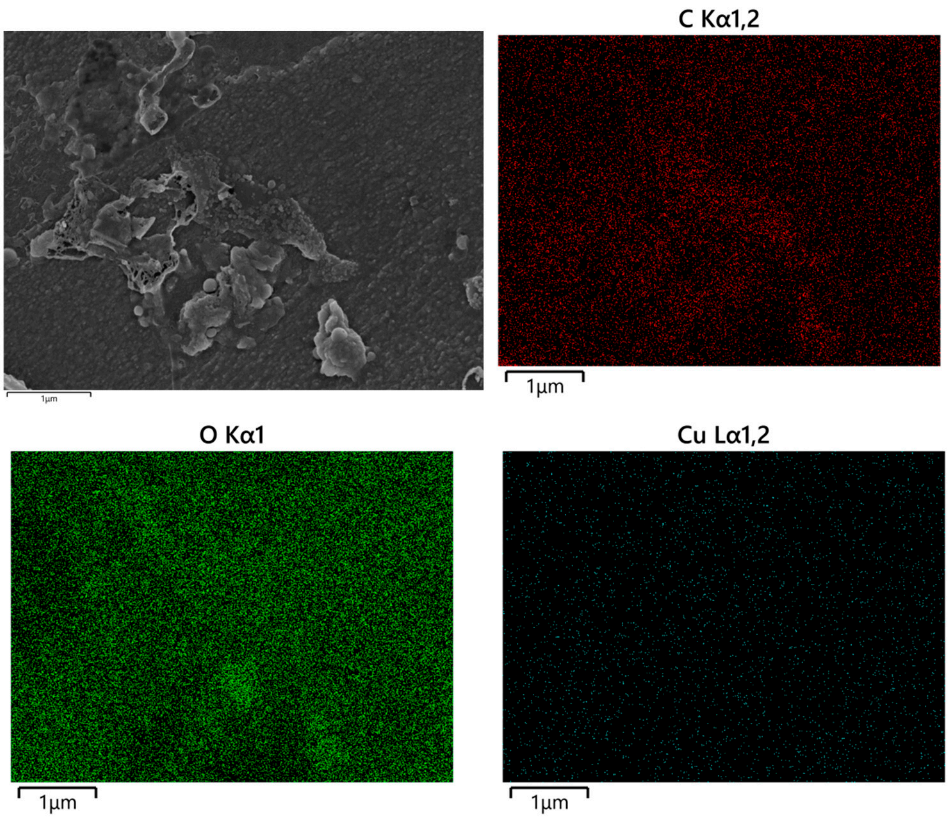

a

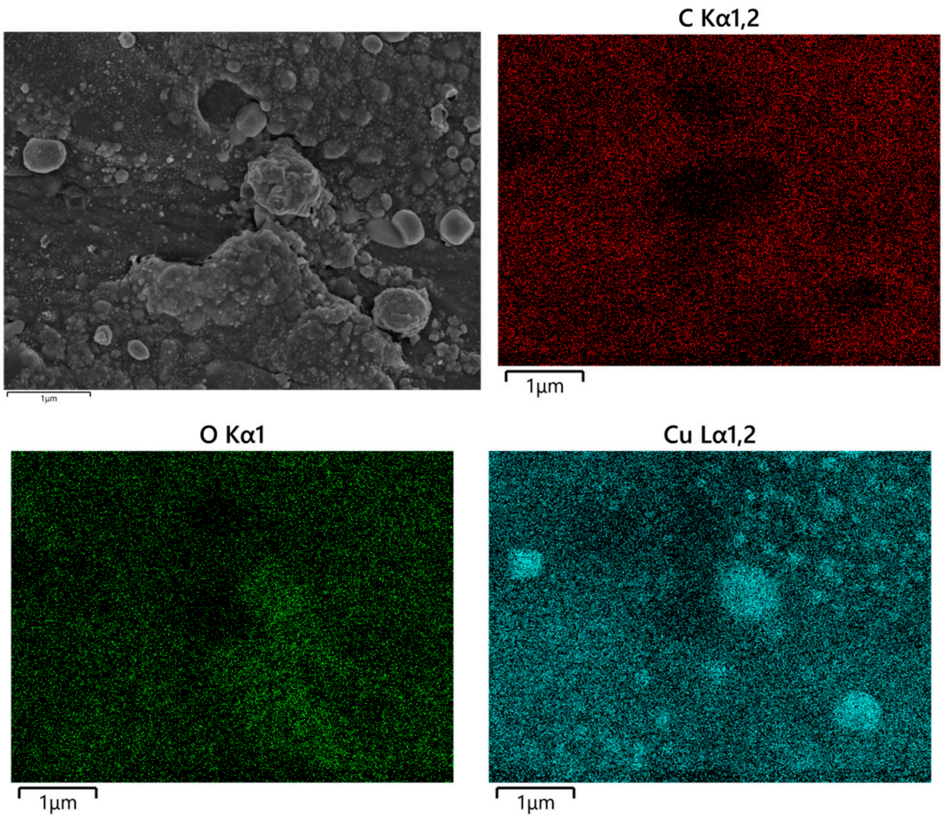
C Kα1,2
1µm
1µm
O Kα1
Cu Lα1,2
1µm
1µm

b

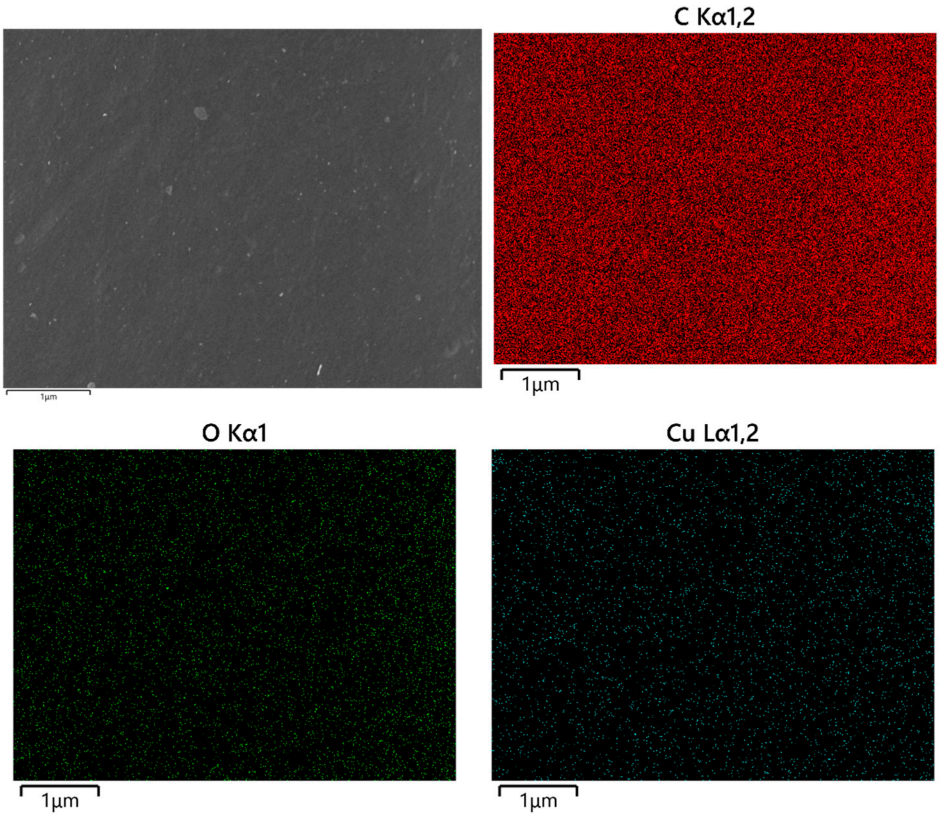
C Kα1,2
1µm
1µm
O Kα1
Cu Lα1,2
1µm
1µm

c

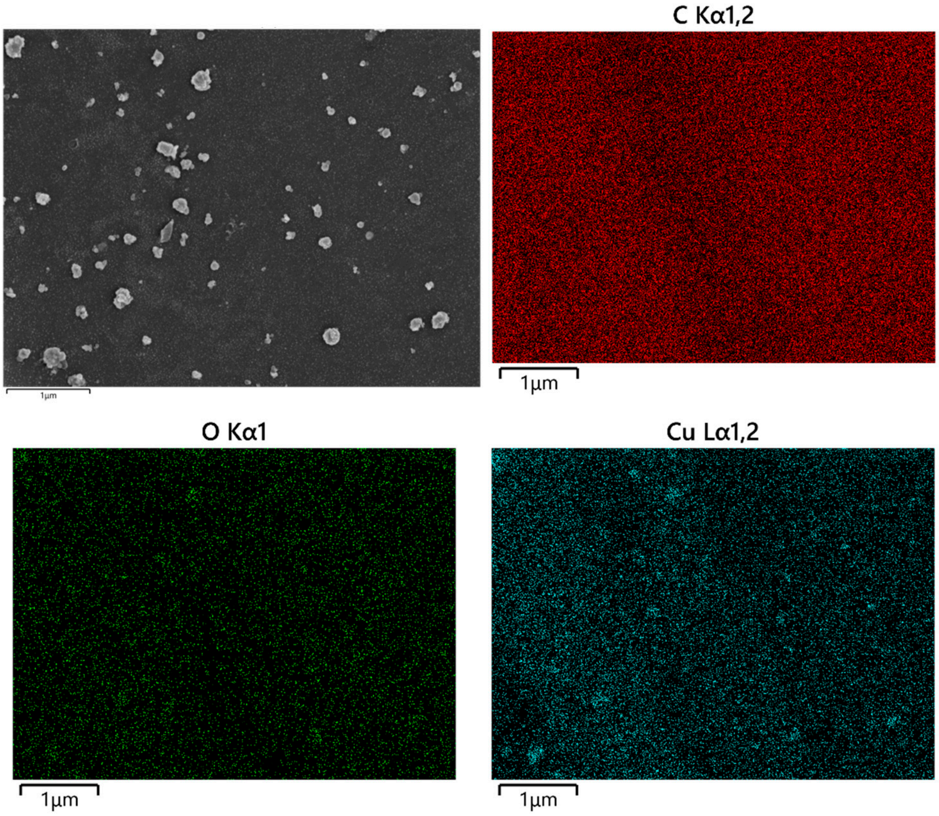
1µm
C Kα1,2
1µm
O Kα1
Cu Lα1,2
1µm
1µm

d

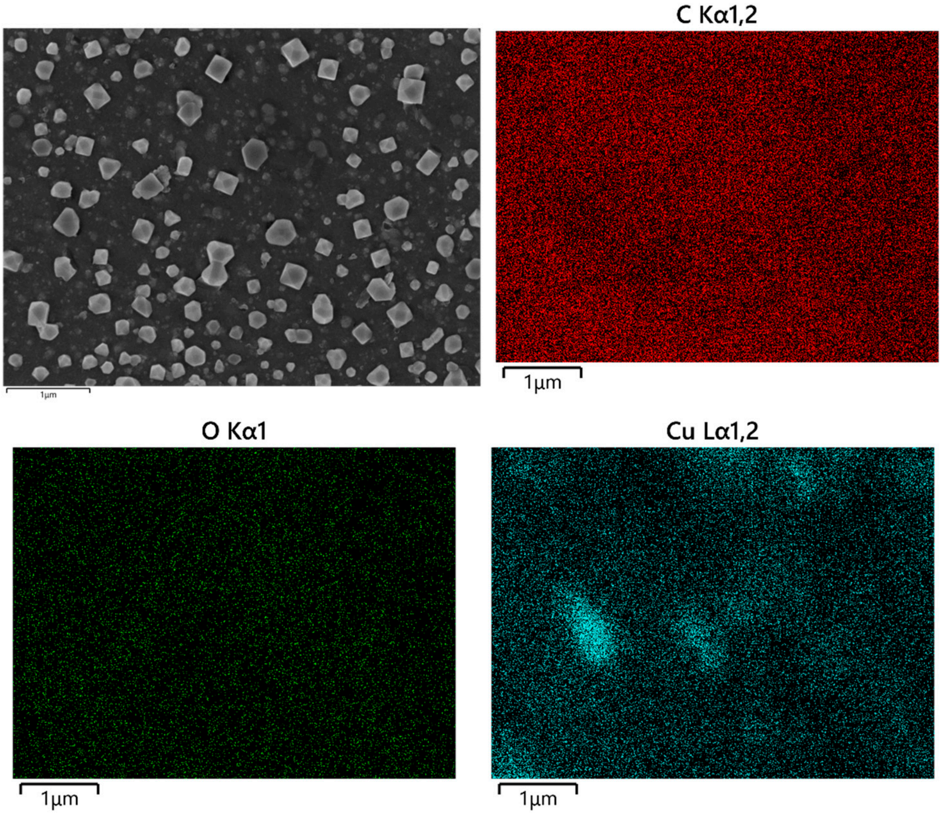
1µm
C Kα1,2
1µm
O Kα1
Cu Lα1,2
1µm
1µm

e

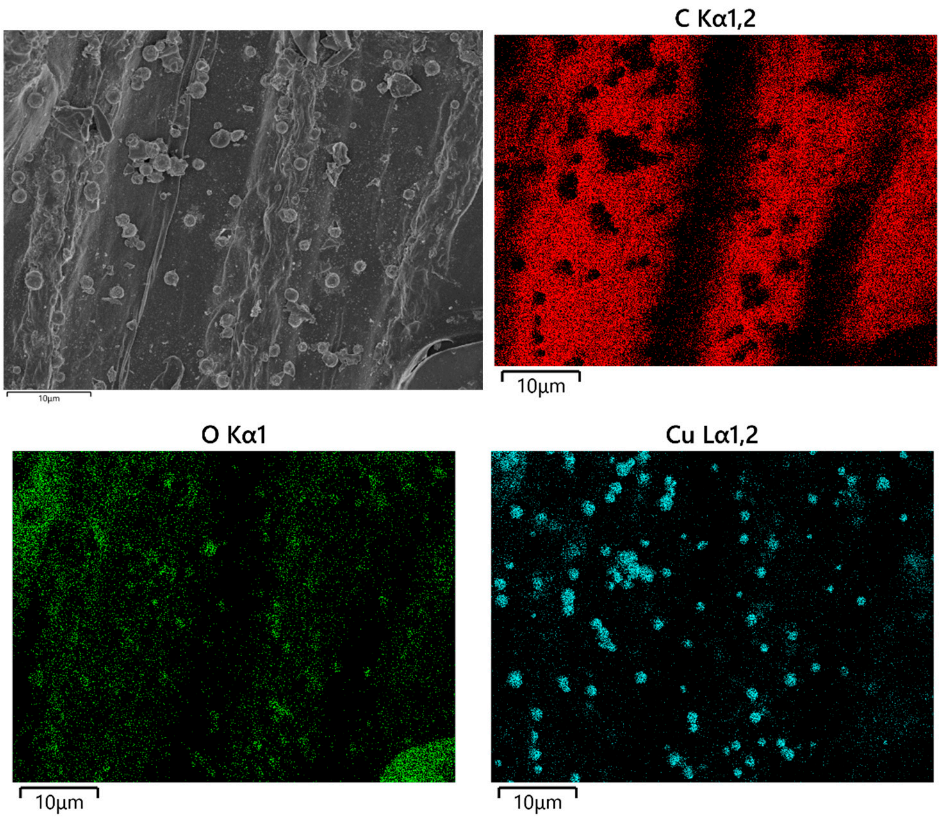

f

SI Figure S2

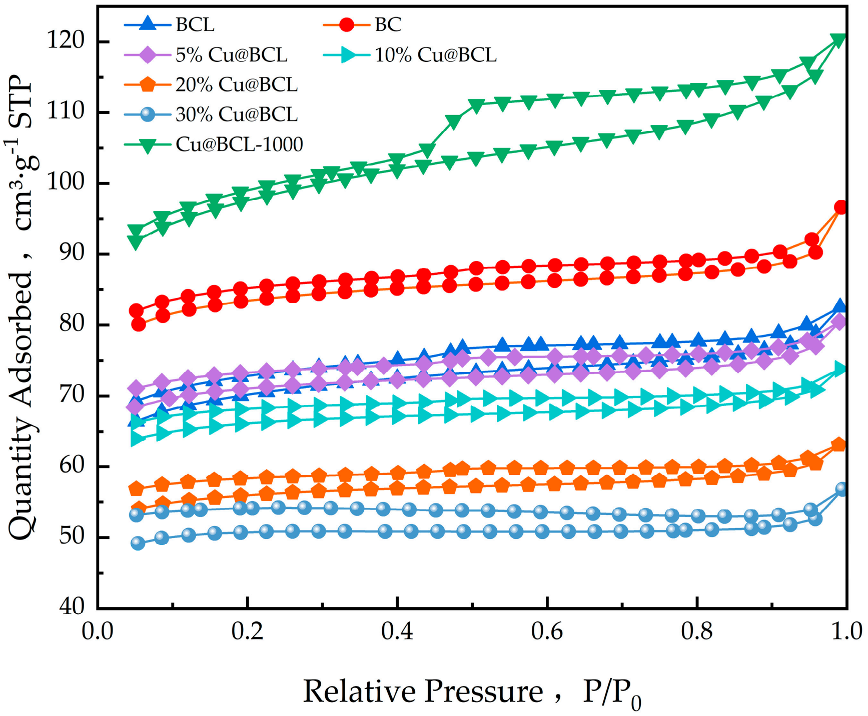

SI Figure S3

SI Table S1

Catalysts	Cu@BCL-600	Cu@BCL-700	Cu@BCL-800	Cu@BCL-900	Cu@BCL-1000
Crystallite size, nm	24.6	25.4	25.8	26.5	26.5

SI Table S2

Sample	BC	Cu@BC	BCL	10%Cu@BCL-700	10%Cu@BCL-800	10%Cu@BC-1000
Cu content	1.21%	25.50%	1.05%	14.94%	15.77%	21.72%
